# Validation of a prediction model for postpartum hospital use in geographic contexts with greater rural representation

**DOI:** 10.1016/j.ajogmf.2026.101959

**Published:** 2026-04-02

**Authors:** Kimberly B. Glazer, Sarah Lindley, Molly Passarella, Teresa Janevic, Natalia Egorova, Jennifer Zeitlin, Scott A. Lorch, Elizabeth A. Howell

**Affiliations:** Department of Obstetrics and Gynecology, University of Pennsylvania Perelman School of Medicine, Philadelphia, PA (Glazer); Department of Biostatistics, Epidemiology, and Informatics, University of Pennsylvania Perelman School of Medicine, Philadelphia, PA (Glazer); Department of Obstetrics and Gynecology, University of Pennsylvania Perelman School of Medicine, Philadelphia, PA (Lindley); Roberts Center for Pediatric Research, Children’s Hospital of Philadelphia, Philadelphia, PA (Passarella); Department of Epidemiology, Mailman School of Public Health, Columbia University, New York, NY (Janevic); Department of Population Health Science and Policy, Icahn School of Medicine at Mount Sinai, New York, NY (Egorova); Université Paris Cité and Université Sorbonne Paris Nord, INSERM, INRAE, Centre for Research in Epidemiology and Statistics, Paris, France (Zeitlin); Department of Pediatrics, University of Pennsylvania School of Medicine, Philadelphia, PA (Lorch); Leonard Davis Institute of Health Economics, Wharton School, University of Pennsylvania, Philadelphia, PA (Lorch); Department of Obstetrics and Gynecology, University of Pennsylvania Perelman School of Medicine, Philadelphia, PA (Howell); Leonard Davis Institute of Health Economics, University of Pennsylvania Perelman School of Medicine, Philadelphia, PA (Howell)

**Keywords:** maternal health, postpartum, prediction, readmission, validation

## Abstract

**BACKGROUND:**

The postpartum period is a critical window to address maternal health inequities. Black, Hispanic, Indigenous, and rural populations experience disproportionately high rates of postpartum morbidity and postpartum hospital use (PHU), defined as readmissions or emergency department (ED) visits after delivery. Delivery hospitalizations provide an opportunity for early identification of individuals at high risk of PHU, who may benefit from targeted interventions to prevent adverse outcomes. We previously developed a 30-day PHU prediction model using New York City (NYC) birth data (2016–2018), which achieved an area under the receiver operating curve (AUC) of 0.69. However, its performance in obstetric populations outside of a dense urban setting has not been examined.

**STUDY DESIGN:**

We aimed to evaluate the accuracy of our PHU prediction model in South Carolina (SC) and Florida (FL), states with diverse populations, including substantial rural representation, and in a different US geographic region than the NYC development sample. We additionally examined model performance in subgroups defined by race/ethnicity, Medicaid insurance, and rural residence.

**METHODS:**

We performed a retrospective cohort study of linked birth certificate and hospital discharge data from 2016 to 2019 births in SC (*n*=183,836) and FL (*n*=696,963). We ascertained 21 predictors consistent with the NYC model, excluding two variables (prenatal depression, Apgar) unavailable in the new states. PHU was defined as ≥1 inpatient or ED encounter within 30 days postpartum. Model performance was assessed using calibration (intercept, slope) and discrimination (AUC). We first applied the original NYC model coefficients to generate PHU predicted probabilities among SC and FL births. We then tested a series of stepwise model updating strategies: recalibrating intercepts, re-estimating predictor coefficients, and incorporating additional contextual indicators of hospital access—residential rurality and driving distance to the delivery hospital—hypothesized to be relevant in settings with larger rural populations.

**RESULTS:**

Cumulative 30-day PHU incidence was 7.4% in SC and 7.2% in FL; rates were higher among Black individuals, Medicaid-insured individuals, and rural residents. Applying the original NYC model coefficients achieved an AUC of 0.68 (95% CI 0.67–0.68) and 0.69 (95% CI 0.68–0.69) among SC and FL births, respectively, but generated overestimated and extreme risk predictions compared with observed risk. Updating model intercepts corrected calibration, and additionally re-estimating coefficients resulted in an AUC of 0.69 (95% CI 0.68–0.69) in SC and 0.70 (95% CI 0.70–0.71) in FL. Inclusion of hospital distance and rurality did not meaningfully change calibration or discrimination. Model discrimination was slightly lower when subset to Black, Medicaid-insured, and rural residents, but AUC increased within each group after re-estimating predictor coefficients.

**CONCLUSION:**

A PHU prediction model developed in an urban NYC cohort demonstrated similarly moderate discrimination in SC and FL as in the original NYC sample, but overestimated absolute risk in these new settings. Modest model updating, including recalibration of intercepts and reestimation of coefficients, yielded well-calibrated models without requiring new predictors. Hospital access measures did not substantially improve prediction. These findings demonstrate that an existing prediction model for postpartum acute care use can be adapted for use in geographically and socio-demographically diverse populations. Geographic validation and model updating are important steps in deploying predictive tools to reduce persistent gaps in maternal health outcomes.

## Tweetable statement

A model developed to predict postpartum hospital use in NYC performed well in states with larger rural populations after modest updates. Geographic validation is a critical step in deploying prediction tools.

## Introduction

More than half of maternal deaths occur in the postpartum period, making the days, weeks, and months following childbirth critical for maternal health. It is also a vital window to redress maternal health inequities, as Black, Hispanic, Indigenous, and rural populations experience higher rates of postpartum morbidity.^1–3^ One of the ways these disparities are reflected is in higher rates of postpartum hospital use (PHU), or readmissions and emergency department (ED) visits after childbirth, among people of color and rural individuals.^2,4^ PHU provides a marker of potentially preventable maternal morbidity, and identifying those at highest risk of unexpected hospital encounters can help direct preventive interventions and support to those most in need.

The delivery hospitalization provides an opportunity to screen for PHU risk and stratify individuals for additional postpartum follow-up. We previously developed a prediction model for 30-day PHU using administrative data on delivery hospitalizations in New York City (NYC) from 2016 to 2018.^4^ The model included 21 predictor variables and achieved an area under the receiver operating curve (AUC) of 0.69 in training, test, and validation data sets. When simulating use of the prediction model paired with a hypothetical postpartum support intervention, we found that identifying high-risk individuals with the prediction model resulted in a greater reduction in racial and ethnic disparities in PHU compared to a conventional approach to identifying high-risk individuals with common pregnancy complications. However, the model was developed from obstetric patients in a densely populated, urban environment with a high concentration of delivery hospitals and tertiary medical centers. It remains unclear whether performance would be adequate in alternate geographic and care settings. Evaluating model performance in external populations is critical to understand its utility in targeting additional sociodemographic disparities, such as the increased morbidity and mortality risks for postpartum individuals in rural US communities.

The objective of the present study was to conduct a geographic validation of our PHU prediction model, previously developed and internally validated in an urban NYC population. We aimed to assess prediction model accuracy when applied to obstetric populations in more diverse geographic settings, selecting South Carolina (SC) and Florida (FL) as states that provide access to large and heterogeneous populations, including substantial numbers of rural residents. Within each state, we also evaluated model performance in the following subgroups: Black individuals, Hispanic individuals, Medicaid-insured individuals, and those residing in rural areas. Finally, because model performance may decline in new geographic or healthcare delivery settings, we examined the impact of a series of model updating steps. Specifically, we tested whether accuracy improved when re-estimating model parameters and incorporating additional indicators of healthcare access—rurality of the maternal residence and distance to the delivery hospital—which we hypothesized would be relevant in states with larger rural populations compared to our NYC development sample.

## Materials and methods

We conducted a retrospective cohort study of linked birth certificate and hospital discharge data from SC and FL from 2016 through 2019. Data linkage was completed by state entities using a combination of iterative deterministic and probabilistic approaches. Linkage was >94% for both states. We included individuals with a delivery hospitalization and discharge date between January 1, 2016, and November 30, 2019, to allow 30 days of postpartum follow-up for all patients. Our study sample included *n*=183,836 deliveries among individuals in SC hospitals and *n*=696,963 in FL hospitals. The study was approved by the Icahn School of Medicine at Mount Sinai Program for the Protection of Human Subjects and the University of Pennsylvania Human Research Protections Program. We followed the Transparent Reporting of a multivariable prediction model for Individual Prognosis or Diagnosis (TRIPOD) Statement guidelines for model validation and reporting.^5^

### Predictor variables

The previously developed model included 21 indicator variables ascertained from NYC birth certificates linked to hospital discharge abstracts with ICD-10-Clinical Modification diagnosis and procedure codes from the delivery hospitalization. We used four statistical and machine learning algorithms to predict PHU, with the final NYC model selected using LASSO (least absolute shrinkage and selection operator) logistic regression. For the present validation study, we ascertained predictor variables in both states as described previously in the model development study (Supplemental Table S1).^4^ The following variables were identified in birth certificates: parity (nulliparous, multiparous), multiple gestation (singleton vs multiple), prepregnancy body mass index (categorized from self-reported height and weight: underweight [<18.5 kg/m^2^], normal weight [18.5 to <25], overweight [25 to <30], class I or II obesity [30 to <40], and class III obesity [≥40]), delivery type (vaginal vs cesarean delivery), gestational age at delivery (<32, 32 to <34, 34 to <37, 37 to <39, ≥39 completed weeks), Apgar score (0–6 vs 7–10), payor (Medicaid, private, uninsured, other payor), and late prenatal care initiation (later than the first trimester or never). The NYC model included prenatal depression and Apgar score, but we did not have information on prenatal depression (SC and FL) and Apgar (SC only) for this validation study.

We used participant zip code to assess area-level measures of structural and social determinants of health (SSDOH), including the Index of Concentration at the Extremes (ICE)^6^ and Child Opportunity Index (COI),^7^ both publicly available. ICE is an area-level indicator of racial-economic segregation that measures the proportion of wealthy White households relative to poor Black households in a zip code. ICE is a continuous measure ranging from −1 to 1, with −1 representing zip codes with entirely poor, Black households and 1 a zip code with entirely wealthy, White households. The COI social/economic environment (SE) index is a score comprised of the neighborhood unemployment rate, poverty rate, commute duration, public assistance rate, home-ownership rate, high-skill employment, median household income, and singleheaded households. In our NYC model development study, we found that ICE and COI SE were interchangeable with respect to predictive performance, and we used the COI SE to measure arealevel SSDOH in SC and FL updated models.

We identified prenatal admissions and ED visits, comorbidities, and obstetrical complications in hospital discharge abstracts. We used ICD-10 diagnosis and procedure codes to classify comorbidities based on Leonard et al,^8^ severe maternal morbidity using the algorithm from the Centers for Disease Control and Prevention,^9^ and obstetrical complications following algorithms from the Joint Commission specifications for perinatal quality measures,^10^ as in the model development and internal validation study.^4^

We ascertained two indicators of hospital access that were not evaluated in model development, and which we hypothesized would improve prediction of PHU outside of the densely populated NYC context. We measured driving distance to the delivery hospital using the distance from the maternal population zcta (zip code tabulation area) centroid to the delivery hospital and created a dichotomous variable for drive time ≥30 minutes. We measured rurality based on maternal residential county from the address reported on the birth certificate and dichotomized using 2024 US Department of Agriculture Urban Influence Codes,^11^ with metropolitan and micropolitan counties defined as urban and counties defined as rural otherwise.

### Outcome

We identified PHU, defined as at least one inpatient hospitalization or ED visit, by linking delivery records prospectively to maternal hospital records up to 30 days after delivery discharge. We created a dichotomous indicator variable for individuals with at least one PHU event during follow-up (PHU=1) and those without any PHU record (PHU=0).

### Statistical analyses

We examined sociodemographic, medical, and obstetric characteristics of participants in each state. Missing data on key covariates was 2.9% overall in SC and 16.0% in FL. Missingness in FL was driven by trimester of prenatal care initiation (11.0% missing) and BMI (5.0%).

We first applied the internally validated (NYC) prediction model to the SC and FL datasets without re-estimating model parameters, using the NYC model coefficients to calculate the predicted probability of PHU for each individual in our study sample. The equation of the NYC PHU prediction model is:

Equation 1. Prediction model for PHU, NYC birth records, 2016 to 2018.^4^

We excluded coefficients for depression (SC and FL) and Apgar score (SC only) from calculation of PHU predicted probabilities, given lack of data on these variables.

We evaluated model performance through calibration and discrimination,^12^ separately for SC births and FL births. Given our objective to evaluate model performance in diverse populations, we additionally ran all models among non-Hispanic Black individuals, Hispanic individuals, Medicaid-insured individuals, and rural residents in each state. We generated decile calibration plots for graphical assessment of model specification and agreement between estimated and observed events. We divided participants into 10 groups based on deciles of predicted probabilities from the logistic regression model and compared the mean predicted probability and mean observed event rate within each decile group. Perfect agreement is represented by the identity line y=x in the plot.^12^ We regressed the observed outcome on predicted probabilities to evaluate mean calibration and calibration slope. Mean calibration, or “calibration-in-the-large,” is represented by the intercept a of the regression equation. The target value is *α*=0 for perfect agreement; *α*<0 suggests the model systematically overestimates PHU risk, and *α*>0 suggests underestimation. Calibration slope measures the spread of estimated risks, with a target value of *β*=1, *β*<1 indicating that model estimated risks are too extreme, and *β*>1 indicating estimated risks are too moderate.^13^ Discrimination, or the ability of the model to separate individuals with a PHU event from those without, was evaluated from the AUC.^14^


Logit(PHU)=−3.8345+0.0385(gestationaldiabetes)+0.384(chronichypertension)+0.2173(preeclampsia)+0.285(gestationalhypertension)+0.1279(asthma)+0.1348(underweight)+0.1942(overweight)+0.3139(class1obesity)+0.5346(class2or3obesity)+0.2181(digestivedisease)+0.4183(majormentalhealthdisorder)+0.3752(neuromusculardisease)+0.0974(laborinduction)+0.0978(fetaldistress)+0.1897(gestationalage<32weeks)+0.2225(gestationalage32to<34wks)+0.0653(gestationalage34to<37wks)+0.0763(gestationalage37to<39wks)+0.6232(prenatalEDvisitoradmission)+0.0967(prenataldepression)+0.4235(severematernalmorbidity)+0.4519(Medicaidinsurance)+−0.046(uninsured)+0.553(otherinsurance)+−0.1433(multiparous)+0.4696(cesareandelivery)+0.1261(Apgarscore)+0.0682(lateprenatalcareinitiation)+−0.4353(IndexofConcentrationattheExtremes)

We next conducted a series of model updating analyses^15^ to tune parameters and compare accuracy of PHU predictions in SC and FL to that achieved using the previously developed NYC model coefficients. First, we recalibrated the model in each state by updating the intercept to allow for differences in baseline PHU risk. Second, we re-estimated all regression coefficients to accommodate potential differences in predictoroutcome associations. Finally, we tested inclusion of indicators of hospital access using driving distance to the delivery hospital and rurality of the maternal residence as two new predictors. We replicated analyses of calibration and discrimination as described previously in each of these model updating steps.

Finally, we re-ran variable selection using LASSO logistic regression with all candidate variables and compared model predictors and AUC resulting from variable re-selection. All analyses were conducted in SAS version 9.4 (SAS Institute, Cary, NC).

## Results

The SC birthing population (*n*=183,836) was 56.9% non-Hispanic White, 30.9% non-Hispanic Black, 4.5% Hispanic, and 1.4% non-Hispanic Asian. In FL (*n*=696,963), a smaller percentage of births were to Black individuals (17.0% of all births) and larger proportions to Hispanic (34.3%), and Asian (3.1%) individuals (Table 1). Rates of private and Medicaid insurance coverage were similar in both states. Roughly 3% (*n*=22,813) of FL birthing individuals and 14% (*n*=25,958) in SC resided in rural areas.

The 2016 to 2019 cumulative incidence of PHU was 7.4% (*n*=13,585) among births in SC and 7.2% (*n*=50,004) in FL (Table 2). Similar patterns in PHU incidence were observed among subgroups in each state, with higher rates among Black individuals (roughly 10%) and those with Medicaid insurance or rural residents (9%) compared to the overall population.

As a first step in geographic validation of the NYC model, we visualized agreement between the predicted PHU probabilities for SC and FL births, calculated using the NYC model coefficients, and observed event rates. On average, the model generated higher PHU predicted probabilities compared to the observed risks, particularly in higher deciles of risk prediction (Figure). Calibration slope was 0.64 in SC and 0.63 in FL, suggesting that predicted risks were more extreme compared to observed risk. The model achieved an AUC of 0.68 (95% CI 0.67 –0.68) and 0.69 (95% CI 0.68–0.69) in discriminating PHU among SC and FL births, respectively (Table 3). AUC decreased when models were run separately among Black, Hispanic, Medicaid-insured, and rural residents.

Model updating improved calibration in both states (Figure), with substantial improvement after adjusting model intercepts (Figure, panels B [SC] and F [FL]) and further incremental benefit from parameter re-estimation and inclusion of new predictors (panels C and D [SC] and G and H [FL]). Models were similarly well-calibrated when we used either driving distance to the delivery hospital, rurality of the maternal residence, or both as indicators of hospital access. In terms of discrimination (Table 3), adjusting the model intercept did not change AUC in either state (Model 1). Re-estimating all predictor coefficients achieved AUC=0.69 (95% CI 0.68–0.69) in the total SC population and 0.70 (95% CI 0.70–0.71) in FL (Table 3, Model 2), as well as slight improvements among Black, Hispanic, Medicaid-insured, and rural residents. We did not observe changes in model discrimination in either state with the inclusion of driving distance to the delivery hospital or rurality of the maternal residence as new predictor variables (Model 3). Reselecting candidate variables produced some variation in the predictors retained in each state’s final model, with driving time to the delivery delivery hospital selected in both, but AUC remained unchanged (Supplemental Table S2).

## Comment

### Principal findings

In this geographic validation study, we found that a model developed to predict PHU among NYC births achieved similar discrimination when applied to obstetric populations in SC and FL. However, calibration was poor and indicated systematic overprediction of PHU risk. Adjusting model intercepts yielded well-calibrated models, and additionally re-estimating predictor coefficients for variables included in the original prediction model increased the AUC to match NYC model discrimination in SC and slightly exceed it in FL. Including indicators of residential distance to delivery hospitals and rurality did not notably improve model calibration or discrimination.

### Results in the context of what is known

Our study contributes to a growing literature on predicting postpartum morbidity, such as hemorrhage,^16^ infection after cesarean delivery,^17^ venous thromboembolism,^18^ and both overall^19^ and cardiovascular^20^ severe maternal morbidity. Specific to PHU, Wen et al^21^ developed a model using California birth certificate and discharge data to predict 60-day ED visits and readmissions, achieving an AUC of 0.67. This performance is comparable to but slightly lower than that reported in internal validation of our NYC model (AUC=0.69).

Despite these advancements, few studies have assessed how postpartum prediction models perform in external populations. To our knowledge, this is the first geographic validation study of a prediction model for acute care utilization in the postpartum period. Applying the original model coefficients yielded comparable discrimination as observed in the development sample but poorer calibration in the new validation states. Discrimination, or the model’s ability to accurately rank individuals at higher vs lower risk, indicates how well the model classifies individuals who will and will not have the outcome of interest.^13^ Calibration refers to how closely the absolute predicted probabilities that the model generates match the observed outcome probabilities. A poorly calibrated model may systematically over- or under-estimate risk but still accurately separate high- and low-risk individuals. Model calibration is often worse in external validation and may result from: (1) overfitting to the development data; (2) differences in baseline outcome prevalence between development and validation samples; (3) variation in predictor-outcome associations across populations; and (4) differences in predictor measurement across datasets.^13,22^ Calibration slope in each state indicated extreme predicted probabilities and model overfitting. Baseline PHU prevalence was higher in both SC parameters improved the accuracy of and FL than in NYC; updating the absolute risk predictions, as reflected in model intercepts and re-estimating calibration plots. In contrast, discrimination statistics such as th AUC, which assess rank ordering of ris estimates to classify risk and are insensi tive to systematic over- or underestimation,^23^ showed only modest chang across our model updating steps.

### Clinical implications

This study has important implication for deploying prediction models in obstetric practice. Health system increasingly look to artificial intelligence to bridge maternal care gaps, such as using electronic health recor (EHR)-embedded predictive analytic to identify high-risk patients for additional monitoring and support.^24^ Thes tools offer the potential to improve allocation of scarce resources and preven excess morbidity and mortality, particularly in the postpartum period whe clinical contact is limited, and warning signs may go undetected. However, geographic differences in population composition, care delivery systems, and clinical practices, and baseline risk may undermine prediction model performance and clinical utility. External validation and calibration across diverse populations and settings are essential to achieve prediction model accuracy and equitable implementation.^15^ Slightly lower discrimination among Black, Hispanic, Medicaid-insured, and rural populations aligns with and extends our previous findings of differential performance across racial and ethnic groups^4^ to additional priority populations. Developing separate models for specific subgroups may contribute to further care fragmentation and risk exacerbating inequities. Instead, our findings highlight the need for ongoing evaluation and updating of prediction models to ensure equitable performance and implementation across populations and settings.

Understanding performance in rural contexts is particularly vital, given that limitations in healthcare access in rural settings are critical determinants of both maternal and infant health outcomes.^25,26^ Barriers to high-quality, timely maternal healthcare in rural areas include lack of risk-appropriate perinatal care facilities; unreliable transportation; limited workforce capacity including availability of obstetric specialists, staff training, and turnover; and care fragmentation.^27–30^ These factors may contribute to differences in model performance across settings but are challenging to operationalize within clinically deployable prediction models. A recent national study found that the number of US hospitals with an obstetric service declined in nearly all states between 2010 and 2022, with losses more pronounced in rural vs urban areas.^31^ In SC, more than one-quarter of hospitals with obstetric services closed their units during this period, and in FL, two-thirds of rural hospitals lacked an obstetric service. Geographic validation of our prediction model showed comparable discrimination to the NYC development cohort without model updating, and good calibration after modest parameter adjustment, suggesting that our PHU model transported well from an urban cohort to two states with more heterogenous healthcare landscapes. We incorporated measures of driving time to the delivery hospital and rurality of the maternal residence as novel indicators of healthcare access, hypothesizing that these variables would be important predictors of PHU in areas with lower population and hospital density.^32,33^ Contrary to our expectation, their inclusion did not change model performance. However, while hospital driving distance did not explain additional variation in PHU beyond existing sociodemographic and clinical predictors included in the original model, its retention in both SC and FL model re-selection suggests that geographic access is a relevant predictor of postpartum acute care needs in these settings. Increased residential distance to maternity care has been associated with greater adverse perinatal outcomes^34,35^ and may be an important contextual factor for intervention design. At the same time, proxies such as rurality and driving distance likely incompletely capture multidimensional access limitations in rural settings.^30^ Key factors, such as care fragmentation, workforce shortages, and transportation availability, are difficult to measure in routinely collected administrative data and may not be readily incorporated into bedside prediction models. Future research should consider how to better measure these dimensions and adapt models to reflect structural access constraints while maintaining clinical usability.

### Research implications

Model updating is a critical but underutilized step in the continuum of prediction model development, validation, deployment, and assessment.^36^ Beyond addressing contextual differences across populations, model updating allows for pragmatic adjustments related to data availability, consistency, and quality that affect model transportability. Although data sources such as hospital claims and EHR enable the inclusion of numerous and detailed predictor variables, ascertainment and completeness often differ across data sources and settings.

Some predictors from our original NYC model were not available in SC or FL datasets, which may contribute to transportability bias if these variables capture risk pathways that differ across populations or care settings. However, rather than deriving entirely new models, updating the existing model retained predictive information gained from the development sample while adapting to data availability and contextual variation in the new prediction tasks.^15^ We explored multiple updating methods in each state validation sample: (1) recalibrating model intercepts to reflect differences in baseline PHU risk, (2) re-estimating all regression coefficients, and (3) including new indicators of population density and hospital access. Our findings, therefore, demonstrate a flexible and dynamic approach to maintaining performance in obstetric risk prediction, in which models can be evaluated and updated over time to remain clinically relevant and equitable across varied health systems and diverse patient populations.^36^

Finally, consistent with prior work,^37^ postpartum readmissions and ED visits were challenging to predict. Our iterative model updating approach yielded minimal gains in AUC, with discrimination similar to that reported in other models of acute postpartum healthcare utilization.^21^ We acknowledge that PHU is a heterogeneous outcome, and that ED visits and readmissions may reflect distinct individual- and systemlevel factors influencing unplanned use of postpartum care, such as differences in severity, access, and care-seeking behaviors. However, in the model development study, we chose to combine ED visits and readmissions into a single outcome as a marker of acute postpartum care needs and potentially preventable maternal morbidity.

In our previous research, we found that predicting specific causes of PHU as opposed to a composite “any cause” outcome yielded higher AUC, but that use of the composite PHU model had the greatest impact on postpartum racial-ethnic inequities when paired with a hypothetical intervention. Additional research, such as decision analyses and impact evaluations comparing the net benefit of using these PHU prediction models with novel care strategies, is warranted to guide clinical implementation. For example, integrating interventions—such as postpartum navigation,^38^ telehealth expansion,^39^ remote monitoring,^40^ and community-based follow-up^41^—with prediction models may enable targeted identification of individuals at highest risk postpartum and facilitate linkage to support services, particularly for populations such as Medicaid-insured or rural-dwelling individuals facing barriers to access and continuity of care.

### Strengths and limitations

Our study was limited by differences in the availability of some data elements across states and potential coding variation in hospital claims data, possibly influencing ascertainment of predictors and outcome variables. We did not have primary collected patient data, such as blood pressure or biomarkers. The modest discrimination observed in our models may reflect limitations in capturing unmeasured social factors and care processes in secondary data sources, such as access to outpatient care and care coordination, suggesting the need for scalable SDOH and obstetric risk screening approaches that can be integrated into clinical models. We were unable to identify obstetrical triage visits, and our estimates of ED use may underestimate care-seeking for complications postpartum. Zip-code-based measures of area deprivation may limit spatial precision^42^ but remain widely used proxies when finer data are unavailable. We did not evaluate the clinical impact of prediction model use, and future research should estimate the benefit of implementing these models with specific postpartum interventions.

Our study has notable strengths, including use of large, representative datasets from two geographically and socio-demographically disparate US states from our model development sample. We benefitted from inclusion of states with substantial rural populations, which allowed us to investigate rural subgroups not available in NYC. We evaluated key indicators of health care access, such as distance to the delivery hospital, which have been largely absent from previous postpartum prediction algorithms. We examined multiple model updating approaches, offering practical guidance for transportability in real-world settings.

## Conclusion

We updated a PHU prediction model developed in NYC for use in SC and FL, states that differ in regional context, population density, and healthcare access. The updated model was well-calibrated to the new study populations and achieved similarly moderate discrimination in SC and FL as in the original NYC sample, demonstrating that existing prediction models can be adapted for new geographic settings. These findings support model validation and updating as key steps toward implementation of equitable and accurate risk prediction tools in postpartum care.

## Supplementary Material

Glazer et al AJOG MFM Supplement

Supplementary material associated with this article can be found in the online version at doi:10.1016/j.ajogmf.2026.101959

## Figures and Tables

**FIGURE:**
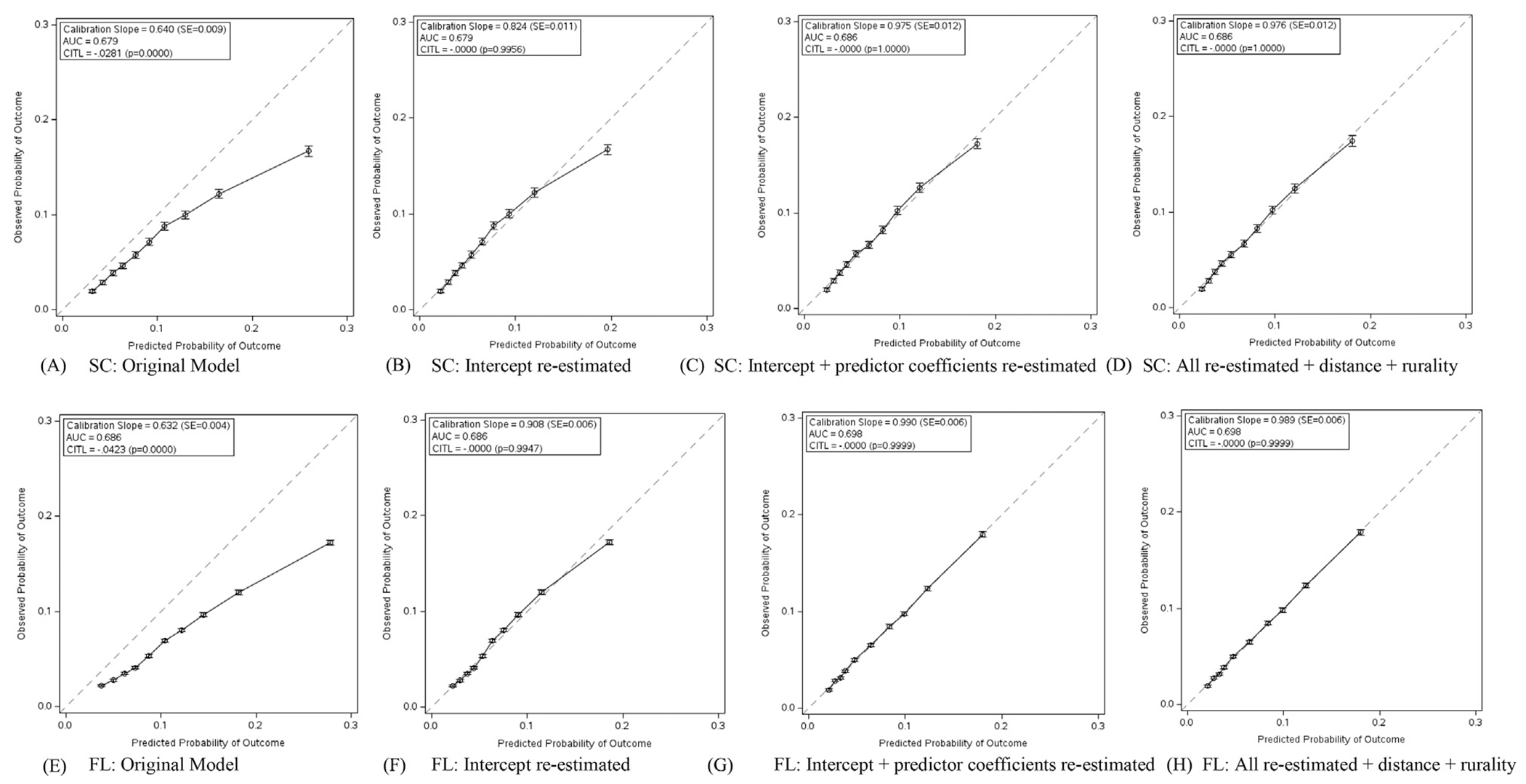
Calibration plots calibration plot shows deciles of mean predicted probability from PHU prediction model compared to mean observed event rate within each decile group. Perfect agreement represented by the identity line *y*=*x*. *CITL*, calibration-in-the-large (CITL; target value *α*=0 indicates perfect agreement, *α*<0 indicates overestimation of risk, and *α*>0 underestimation). Calibration slope measures spread of estimated risks (target value of *β*=1, *β*<1 indicates model estimated risks are too extreme, *β*>1 risks are too moderate). Glazer.Validationofapredictionmodelforpostpartumhospitaluse.AmJObstetGynecolMFM2026.

**TABLE 1:** Characteristics of births in Florida (*n*=696,963) and South Carolina (*n*=183,836) hospitals, 2016 to 2019, and 2016 to 2017 New York City births used in prediction model development^a^

	Florida *n*=696,963	South Carolina *n*=183,836	New York City *n*=145,464
Demographic characteristics			
Maternal age (y)			
<20	31,839 (4.6)	11,928 (6.5)	4178 (2.9)
20–34	535,798 (76.9)	146,475 (79.7)	104,494 (71.8)
35+	129,326 (18.6)	25,433 (13.8)	36,785 (25.3)
Race/ethnicity			
Black, non-Hispanic	11,228 (17.0)	56,866 (30.9)	27,680 (19.0)
Hispanic	239,326 (34.3)	8250 (4.5)	41,764 (28.7)
White, non-Hispanic	297,854 (42.7)	104,608 (56.9)	48,503 (33.4)
Asian, non-Hispanic	21,666 (3.1)	2536 (1.4)	26,902 (18.5)
Other race, non-Hispanic	19,889 (2.9)	11,576 (6.3)	615 (0.4)
Education			
Less than high school	76,409 (11.0)	24,761 (13.5)	24,800 (17.1)
High school	215,280 (30.9)	45,381 (24.7)	32,966 (22.7)
Greater than high school	400,241 (57.4)	113,348 (61.7)	87,349 (60.2)
Unknown or missing	5033 (0.7)	346 (0.2)	0 (0.0)
Insurance			
Medicaid	327,871 (47.0)	84,033 (45.7)	86,827 (59.7)
Private insurance	318,322 (45.7)	81,875 (44.5)	55,958 (38.5)
Uninsured	25,682 (3.7)	3728 (2.0)	1287 (0.9)
Other payor	25,088 (3.6)	14,200 (7.7)	1392 (1.0)
Birth country			
US-born	465,563 (66.8)	Not ascertained	75,632 (52.0)
Non-US-born	231,400 (33.2)	Not ascertained	69,832 (48.0)
Medical and obstetric characteristics			
Body mass index			
Underweight	25,993 (3.7)	6274 (3.4)	7735 (5.3)
Normal weight	302,796 (43.4)	70,618 (38.4)	76,226 (52.4)
Overweight	187,904 (27.0)	46,316 (25.2)	35,884 (24.7)
Class 1–2 obesity	148,141 (21.3)	46,287 (25.2)	21,906 (15.1)
Class 3 obesity	32,129 (4.6)	14,341 (7.8)	3162 (2.2)
Comorbidities			
Chronic hypertension	24,457 (3.5)	10,749 (5.8)	3995 (2.8)
Gestational hypertension	68,583 (9.8)	21,588 (11.7)	10,780 (7.4)
Preeclampsia	42,261 (6.1)	12,515 (6.8)	3483 (2.4)
Diabetes mellitus	7601 (1.1)	3067 (1.7)	1930 (1.3)
Gestational diabetes	60,208 (8.6)	16,093 (8.8)	14,271 (9.8)
Asthma	30,003 (4.3)	9155 (5.0)	20,896 (6.3)
Digestive disease	36,578 (5.2)	12,028 (6.5)	24,735 (7.5)
Major mental health disorder	31,386 (4.5)	14,062 (7.6)	32,575 (9.9)
Neuromuscular disease	10,096 (1.4)	3574 (1.9)	7257 (2.2)
Prenatal depression	Not ascertained	Not ascertained	18,991 (13.1)
Parity			
Nulliparous	281,763 (40.4)	70,669 (38.4)	63,158 (43.3)
Multiparous	415,200 (59.6)	113,167 (61.6)	82,242 (56.6)
Late prenatal care initiation^b^	144,537 (20.7)	48,541 (26.4)	34,786 (23.9)
Prenatal ED visit or admission	306,126 (43.9)	83,071 (45.2)	58,239 (40.0)
Labor induction	115,157 (16.5)	42,855 (23.3)	24,751 (17.0)
Fetal distress	115,157 (16.5)	25,103 (13.7)	24,222 (16.7)
Gestational age at delivery			
<32 completed weeks of gestation	8871 (1.3)	2974 (1.6)	1720 (1.2)
32 to <34 wk	7436 (1.1)	2451 (1.3)	1311 (0.9)
34 to <37 wk	48,408 (6.9)	14,517 (7.9)	8400 (5.8)
37 to <39 wk	189,640 (27.2)	51,239 (27.9)	34,761 (23.9)
≥39 wk	442,608 (63.5)	112,655 (61.3)	99,271 (68.2)
Delivery type			
Vaginal	433,112 (62.1)	121,337 (66.0)	97,726 (67.2)
Cesarean	263,851 (37.9)	62,499 (34.0)	47,737 (32.8)
Apgar score			
0–6	11,894 (1.7)	Not ascertained	6066 (4.2)
7–10	685,069 (98.3)	Not ascertained	139,282 (95.8)
Residential neighborhood characteristics			
ICE, mean (SD)	0.11 (0.16)	0.05 (0.16)	0.11 (0.22)
COI-SE z-score, mean (SD)	−0.04 (0.13)	−0.03 (0.13)	−0.13 (0.20)
Population density			
Rural	22,813 (3.3)	25,958 (14.1)	Not ascertained
Urban	674,141 (96.7)	157,874 (85.9)	Not ascertained
Distance to delivery hospital, median (IQR), min	19.1 (12.7, 27.8)	19.4 (13.2–29.0)	Not ascertained

Data from Florida and South Carolina include births to individuals with a delivery hospitalization discharge date between 1/1/2016 and 11/30/2019. New York City data show the distribution of births in the original prediction model training data set (random sample of 70% of all live births in NYC hospitals with delivery discharge date between 1/1/2016 and 11/30/2017). Rural vs urban residence not assessed in NYC. *COI—SE, Child Opportunity Index, social/economic environment; ICE, Index of Concentration at the Extremes*.

a Training data used for model development included 70% of 2016 to 2017 NYC hospital birth (n=145,464 births).

b After first trimester or never.

Glazer. Validation of a prediction model for postpartum hospital use. Am J Obstet Gynecol MFM 2026.

**TABLE 2:** Thirty-day cumulative incidence of postpartum hospital use in development cohort (New York City births, 2016–2017) and validation cohorts (South Carolina and Florida births, 2016–2019), overall and by sociodemographic characteristics

	New York City^a^		South Carolina		Florida	
	Total *n*	PHU *n* (%)	Total *n*	PHU *n* (%)	Total *n*	PHU *n* (%)
All births	145,464	8291 (5.7)	183,836	13,585 (7.4)	696,963	50,004 (7.2)
Black, NH	27,680	2547 (9.2)	56,866	5885 (10.3)	118,228	12,697 (10.7)
Hispanic	41,764	3132 (7.5)	8250	480 (5.8)	239,326	14,915 (6.2)
Medicaid	86,827	6078 (7.0)	84,033	8146 (9.7)	327,871	29,973 (9.1)
Rural	NA	NA	25,958	2318 (8.9)	22,813	2047 (9.0)

*NH*, non-Hispanic; *PHU*, postpartum hospital use.

a NYC births included in training data from development cohort (70% of all births included in training dataset).

Glazer. Validation of a prediction model for postpartum hospital use. Am J Obstet Gynecol MFM 2026.

**TABLE 3:** Area under the receiver operating curve (95% confidence interval) comparing discrimination of original and updated postpartum hospital use prediction models in South Carolina (*n*=183,836) and Florida (*n*=696,963) hospital births, 2016 to 2019

	NYC Original Model	South Carolina				Florida			
		Original Model	Updated Model 1	Updated Model 2	Updated Model 3	Original Model	Updated Model 1	Updated Model 2	Updated Model 3
All births	0.69	0.68	0.68	0.69	0.69	0.69	0.69	0.70	0.70
		
		(0.67–0.68)	(0.67–0.68)	(0.68–0.69)	(0.68–0.69)	(0.68–0.69)	(0.68–0.69)	(0.70–0.71)	(0.70–0.70)

Black, NH	0.65	0.63	0.63	0.64	0.64	0.65	0.65	0.66	0.66
		
		(0.63–0.64)	(0.63–0.64)	(0.64–0.65)	(0.63–0.65)	(0.64–0.65)	(0.64–0.65)	(0.65–0.66)	(0.66–0.67)

Hispanic	0.65	0.65	0.65	0.67	0.67	0.68	0.68	0.70	0.70
		
		(0.62–0.67)	(0.62–0.67)	(0.64–0.69)	(0.64–0.70)	(0.67–0.68)	(0.67–0.68	(0.70–0.71)	(0.70–0.71)

Medicaid	NA	0.64	0.64	0.65	0.65	0.67	0.67	0.68	0.68
		
		(0.64–0.65)	(0.64–0.65)	(0.65–0.67)	(0.64–0.66)	(0.66–0.67)	(0.66–0.67)	(0.68–0.69)	(0.68–0.69)

Rural	NA	0.65	0.65	0.66	0.66	0.66	0.66	0.68	0.68
		
		(0.64–0.66)	(0.64–0.66)	(0.65–0.67)	(0.65–0.67)	(0.65–0.67)	(0.65–0.67)	(0.67–0.69)	(0.67–0.69)

NYC training data included 145,464 hospital births from 1/1/2016 to 11/30/2017, followed for 30 days postpartum.

*AUC,* area under the receiver operating curve; *Model 1*, re-estimated intercept+NYC predictor coefficients; *Model 2,* re-estimated intercept+NYC predictors with re-estimated coefficients; *Model 3,* Model 2 approach+driving distance to delivery hospital+rurality; *NA*, not assessed.

Glazer. Validation of a prediction model for postpartum hospital use. Am J Obstet Gynecol MFM 2026.

## AJOG MFM at a Glance

### Why was this study conducted?

We previously developed a prediction model that accurately ascertained risk of postpartum readmissions and emergency department visits (“postpartum hospital use”) among New York City births. This study evaluated its performance in South Carolina and Florida, states in a different geographic region with larger rural populations.

### Key findings

The NYC model showed similar discrimination but poor calibration in South Carolina and Florida. Adjusting intercepts corrected calibration, and re-estimating predictor coefficients increased the area under the receiver operating characteristic curve to match NYC model discrimination in South Carolina and slightly exceed it in Florida.

### What does this add to what is known?

A model developed to predict postpartum hospital use in an urban cohort can be adapted for use in other settings with modest updates. Findings highlight the utility of geographic validation and model updating for accurate risk prediction in obstetric care.
