# Supplementary material for: Validation of a prediction model for postpartum hospital use in geographic contexts with greater rural representation
Source: Am J Obstet Gynecol MFM. Author manuscript; Available in PMC 2026 Jul 29. (PMC13417675; doi:10.1016/j.ajogmf.2026.101959)
Supplement: Glazer et al AJOG MFM Supplement [file NIHMS2192911-supplement-Glazer_et_al_AJOG_MFM_Supplement.docx]

**Supplementary Material for “Validation of a prediction model for postpartum hospital use in geographic contexts with greater rural representation”**

Kimberly B. Glazer PhD MPH (ORCID: 0000-0002-3318-9802)^1^, Sarah Lindley PhD

(0009-0001-7638-0869)^2^, Molly Passarella MS (0009-0000-8037-152X)^3^, Teresa Janevic PhD MPH (0000-0002-9934-4406)^4^, Natalia Egorova PhD (0000-0002-5448-0471)^5^, Jennifer Zeitlin DSc MA (0000-0002-9568-2969)^6^, Scott A. Lorch MD MSCE (0000-0003-3855-6752)^7^, Elizabeth A. Howell MD MPP (0009-0004-4835-3240)^8^

^1^ Department of Obstetrics and Gynecology, University of Pennsylvania Perelman School of Medicine, Philadelphia, PA; Department of Biostatistics, Epidemiology, and Informatics, University of Pennsylvania Perelman School of Medicine, Philadelphia, PA

^2^ Department of Obstetrics and Gynecology, University of Pennsylvania Perelman School of Medicine, Philadelphia, PA

^3^ Roberts Center for Pediatric Research, Children's Hospital of Philadelphia, Philadelphia, PA

^4^ Department of Epidemiology, Mailman School of Public Health, Columbia University, New York, NY

^5^ Department of Population Health Science and Policy, Icahn School of Medicine at Mount Sinai, New York, NY

^6^ Université Paris Cité and Université Sorbonne Paris Nord, INSERM, INRAE, Centre for Research in Epidemiology and Statistics, F-75004 Paris, France

^7^ Department of Pediatrics, University of University of Pennsylvania School of Medicine, Philadelphia, Pennsylvania; Leonard Davis Institute of Health Economics, Wharton School, University of Pennsylvania, Philadelphia, Pennsylvania.

^8^ Department of Obstetrics and Gynecology, University of Pennsylvania Perelman School of Medicine, Philadelphia, PA; Leonard Davis Institute of Health Economics, University of Pennsylvania Perelman School of Medicine, Philadelphia, PA

**Contents**

**Table S1.** Postpartum Hospital Use Prediction Model Candidate Variables and Corresponding Source

**Table S2.** Adjusted odds ratios (95% confidence limits) for updated prediction models for postpartum hospital use^a^, South Carolina and Florida hospital births, 2016-2019

**Table S3.** Results of variable re-selection with lasso logistic regression using all candidate variables, South Carolina and Florida, 2016-2019

**Table S1.** Postpartum Hospital Use Prediction Model Candidate Variables and Corresponding Source

| **Candidate Variable** | **ICD-10-CM (Diagnosis Codes)** | **ICD-10-PCS (Procedure Codes)** | **Other Source** |
| --- | --- | --- | --- |
| Preexisting diabetes mellitus | E08x-E13x, O24.0x, O24.1x, O24.3x, O24.8x, Z79.4x |  | Birth Record |
| Gestational diabetes mellitus | O24.4x, O24.9x |  | Birth Record |
| Preexisting/chronic hypertension | O10x, I10x |  | Birth Record |
| Preeclampsia | O11x, O14.0x-O14.2x, O14.9x |  | Birth Record |
| Gestational hypertension | O13x |  | Birth Record |
| Previous cesarean birth | O34.21x |  |  |
| Pulmonary hypertension | I27.0x, I27.2x |  |  |
| Asthma (acute or moderate/severe) | O99.5x, J45.21x, J45.22x, J45.31x, J45.32x, J45.4x, J45.5x, J45.901x, J45.902x, J45909x, J4599x |  |  |
| Other pulmonary disease | J410x, J411x, J418x, J42x, J430x-J432x, J438x-J441x, J449x, J470x, J471x, J479x, J670x-J679x, J60x-J64x, J66x, J840x-J842x, J848x, J849x, J17x, M3481x, J99x |  |  |
| Bleeding disorder, preexisting | D66x-D69x (excluding 68.8x and 68.9x) |  |  |
| BMI |  |  | Birth record |
| Cardiac disease, preexisting | I05x-I09x, I11x-I13x, I15x, I16x, I20x, I25x, I27.8x, I30x-I39x, I40x, I41x, I44x-I49x, I50.22x, I50.23x, I50.32x, I50.33x, I50.42x, I50.43x, I50.812x, I50.813x, O99.41x, O99.43x O99.42x, Q20x-Q24x, O990.3x |  |  |
| Chronic renal disease | O26.83x, I12x, I13x, N03x, N04x, N05x, N07x, N08x, N11.1x, N11.8x, N11.9x, N18x, N25.0x, N25.1x, N25.81x, N25.89x, N25.9x, N26.9x |  |  |
| Connective tissue or autoimmune disease | M30x-M36x |  |  |
| Placental disorders (including placenta previa, complete or partial, placental abruption, and placenta accrete spectrum) | O44.03x, O44.13x, O44.23x, O44.33x, O45x, O43.2x |  |  |
| Substance use disorder | F10x-F19x, O99.31x, O99.32x |  |  |
| Anemia, preexisting | O99.01x, O99.02x, D50x, D55x, D56x, D57.1x, D57.20x-D57.40x, D57.80x, D58x, D59x |  |  |
| Bariatric surgery | O99.84x |  |  |
| Digestive disorders | K00x-K09x, K10x-K19x, K20x-K29x, K30x-K39x, K40x-K49x, K50x-K59x, K60x-K69x, K70x-K79x, K80x-K89x, K90x- K95x, O99.6x, O26.6x |  |  |
| Major mental health disorders | O99.34x, F20x-F29x, F30x, F31x-F39x |  |  |
| Social determinants of health | Z55x-Z65x |  |  |
| Neuromuscular diseases | O99.35x, G40x, G70x, G35-G37 |  |  |
| Thyrotoxicosis | E05x |  |  |
| Congenital anomalies |  |  | Birth record |
| Intrauterine growth restriction | O36.59x |  |  |
| Labor induction | 61.1x | 0U7C7ZZ, 10900ZC, 3E033VJ, 3E0P7GC |  |
| Endometritis | O86.12x |  |  |
| Antepartum/intrapartum hemorrhage | O46.0x, O46.8x, O46.9x, O67.0x, O67.8x, O67.9x |  |  |
| Postpartum hemorrhage | O43.212x, O43.213x, O43.219x, O43.222x, O43.223x, O43.229x, O43.232x, O43.233x, O43.239x, O72.0x-O72.3x |  |  |
| Prelabor uterine rupture | O71.02, O71.03 |  |  |
| Blood complications/antepartum coagulation deficiency | O99.1x , Z79.01 |  |  |
| Polyhydraminos/ oligohydramnios | O41.0x, O40.x |  |  |
| Cord prolapse | O69.0x |  |  |
| Isoimmunization | O36.0x, O36.1x |  |  |
| PROM | O42.0, O42.1, O42.9 |  |  |
| Premature separation of placenta | O45.0x, O45.8x, O45.9x |  |  |
| Amniotic infection/chorioamnionitis | O41.10x, O41.12x, O41.14x |  |  |
| Gestational age |  |  | Birth record |
| Fetal distress | O36.8x, ICD-10-CM O76x |  |  |
| Obstructed labor | O66x |  |  |
| Vasa previa | O69.4x |  |  |
| Labor and delivery complicated by abnormality of fetal acid-base balance | O68 |  |  |
| Severe maternal morbidity (SMM) | 121.x, 122.x, 171.x, 179.0x, N17.x, O90.4x, J80x, J95.1x, J95.2x, J95.3x, J95.82x, J96.0x, J96.2x, R09.2x, O88.1x, I46.x, I49.0x, D65x, D68.8x, D68.9x, O72.3x, O15.x, I97.12x, I97.13x, I97.710x, I97.711x, I60.xx-I68.xx, O22.51x-O22.53x, I97.81x, I97.82x, O87.3x, J81.0x, I50.1x, I50.20x-I50.23x, I50.30x-I50.33x, I50.40x-I50.43x, I50.9x, O74.0x-O74.3x, O89.0x- O89.2x, O85x, O86.04x, T80.211Ax, T81.4XXAx, T81.44XXAx, R65.20x, A40.x, A41.x, A32.7x, O75.1x, R57.x, R65.21, T78.2XXA, T88.2XXA, T88.6XXA, T81.10XA, T81.11XA, T81.19XA, D57.0x, D57.21x, D57.41x, D57.81x, I26.x, O88.0x, O88.2x, O88.3x, O88.8x | 5A2204Z, 5A12012, 0UT90ZZ, 0UT94ZZ, 0UT97ZZ, 0UT98ZZ, 0UT9FZZ, 0B110Z, 0B110F, 0B113, 0B114, 5A1935Z, 5A1045Z, 5A1955Z, 30233H1- 30233T1, 30233H0-30230T1, 30230H0-30240T1, 30240H0-30243T1’, 30243H0-30250T1, 30250H0-30253T1, 30253H0-30253T0, 30260H1-30260T1, 30260H0-30260T0, 30263H1-30263T1, 30263H0-30263T0 |  |
| Alcohol use | O99.31x |  | Birth record |
| Drug use | O99.32x |  | Birth record |
| Tobacco use | O99.33x |  | Birth record |
| Prenatal anxiety disorder | F40.0x-F48.9x |  |  |
| Personal history of other mental or behavioral disorders |  |  | Birth record |
| Intimate partner violence | O9A.4x, T74.11x, T74.21x, T74.31x, T74.91x, T76.11x, T76.21x, T76.31x, T76.91x, Y07.01x, Z04.41x, Z04.71x, Z63.0x, Z91.41x |  |  |
| Maternal age |  |  | Birth record |
| Mother’s education level |  |  | Birth record |
| Insurance |  |  | Birth record |
| Parity |  |  | Birth record |
| Delivery mode |  |  | Birth record |
| Apgar score |  |  | Birth record |
| Plurality (singleton v. multiple birth) |  |  | Birth record |
| Nativity (US-born v. foreign-born) |  |  | Birth record |
| Late entry into prenatal care |  |  | Birth record |
| Health environment |  |  | Child Opportunity Index |
| Social environment |  |  | Child Opportunity Index |
| Structural racism |  |  | Index of Concentration at the Extremes |
| Rurality |  |  | Population density of maternal residential county |
| Access to care |  |  | Driving distance to delivery hospital |

**Table S2.** Adjusted odds ratios (95% confidence limits) for updated prediction models for postpartum hospital use^a^, South Carolina and Florida hospital births, 2016-2019

|  | **South Carolina** | | | **Florida** | | |
| --- | --- | --- | --- | --- | --- | --- |
| **Effect** | **OR** | **LCL** | **UCL** | **OR** | **LCL** | **UCL** |
| Antepartum ED visit or readmission | 2.13 | 2.05 | 2.13 | 2.77 | 2.71 | 2.83 |
| Body Mass Index |  |  |  |  |  |  |
| Underweight | 0.99 | 0.89 | 0.99 | 1.08 | 1.02 | 1.14 |
| Normal weight | Ref | Ref | Ref | Ref | Ref | Ref |
| Overweight | 1.09 | 1.03 | 1.09 | 1.12 | 1.10 | 1.15 |
| Class 1-2 obesity | 1.31 | 1.25 | 1.31 | 1.36 | 1.33 | 1.39 |
| Class 3 obesity | 1.69 | 1.59 | 1.69 | 1.74 | 1.68 | 1.81 |
| Apgar score |  |  |  |  |  |  |
| 0-6 | n/a | n/a | n/a | 1.11 | 1.04 | 1.18 |
| 7-10 | Ref | Ref | Ref | Ref | Ref | Ref |
| Asthma | 1.26 | 1.17 | 1.35 | 1.26 | 1.21 | 1.31 |
| Chronic hypertension | 1.21 | 1.13 | 1.29 | 1.29 | 1.24 | 1.35 |
| Cesarean delivery | 1.46 | 1.40 | 1.52 | 1.48 | 1.45 | 1.51 |
| Digestive disease | 1.25 | 1.17 | 1.33 | 1.21 | 1.17 | 1.26 |
| Driving distance to hospital >30 min | 1.02 | 0.98 | 1.06 | 1.02 | 1.00 | 1.05 |
| Fetal distress | 1.00 | 0.95 | 1.05 | 1.04 | 1.01 | 1.07 |
| Gestational diabetes | 1.00 | 0.94 | 1.06 | 1.02 | 0.99 | 1.05 |
| Gestational hypertension | 1.09 | 1.03 | 1.15 | 1.21 | 1.17 | 1.24 |
| Gestational age at birth (wks) |  |  |  |  |  |  |
| <32 | 1.24 | 1.10 | 1.40 | 1.46 | 1.36 | 1.57 |
| 32-<34 | 1.03 | 0.89 | 1.18 | 1.29 | 1.20 | 1.40 |
| 34-<37 | 1.09 | 1.02 | 1.16 | 1.14 | 1.10 | 1.18 |
| 37-<39 | 1.07 | 1.03 | 1.12 | 1.10 | 1.07 | 1.12 |
| ≥39 | Ref | Ref | Ref | Ref | Ref | Ref |
| Induction of labor | 1.13 | 1.08 | 1.18 | 1.02 | 0.99 | 1.05 |
| Length of stay (delivery stay) | 1.02 | 1.01 | 1.02 | 0.98 | 0.98 | 0.99 |
| Major mental health disorder | 1.39 | 1.31 | 1.47 | 1.36 | 1.31 | 1.41 |
| Multiparity | 0.92 | 0.88 | 0.95 | 0.96 | 0.94 | 0.98 |
| Neuromuscular disease | 1.37 | 1.23 | 1.52 | 1.39 | 1.31 | 1.47 |
| Payor |  |  |  |  |  |  |
| Medicaid | 1.54 | 1.48 | 1.61 | 1.34 | 1.31 | 1.37 |
| Private | Ref | Ref | Ref | Ref | Ref | Ref |
| Uninsured | 1.05 | 0.90 | 1.22 | 0.53 | 0.49 | 0.58 |
| Other payor | 1.44 | 1.34 | 1.54 | 1.33 | 1.27 | 1.40 |
| Preeclampsia | 1.13 | 1.05 | 1.20 | 1.13 | 1.09 | 1.18 |
| Prenatal care initiation>1^st^ trimester | 1.01 | 0.97 | 1.06 | 1.01 | 0.99 | 1.04 |
| Severe maternal morbidity | 1.61 | 1.44 | 1.79 | 1.43 | 1.35 | 1.52 |
| Rural residence | 1.03 | 0.97 | 1.08 | 1.08 | 1.03 | 1.13 |
| COI, social/economic environment | 0.46 | 0.39 | 0.55 | 0.92 | 0.85 | 0.99 |

OR=Odds ratio, LCL=lower confidence limit, UCL=upper confidence limit, wks=weeks; ^a^Model updated with re-estimated intercept and predictor coefficients + additional predictor variables

**Table S3.** Results of variable re-selection with lasso logistic regression using all candidate variables, South Carolina and Florida, 2016-2019

| State | Variables Selected | AUC |
| --- | --- | --- |
| South Carolina | 24 variables:  Chronic hypertension  Preeclampsia  Gestational hypertension  Asthma  BMI  **Substance use disorder**  **Anemia**  Digestive disease  Major mental health disorder  Labor induction  **Postpartum hemorrhage**  **Length of stay**  Antepartum admission or ED visit  **Tobacco use**  **Anxiety**  SMM  **Age**  **Education**  Insurance  Parity  Delivery mode  **COI – SE**  ICE  **Distance to nearest delivery hospital (continuous, minutes)^a^** | 0.69 |
| Florida | 20 variables:  Chronic hypertension Preeclampsia  Gestational hypertension  Asthma  BMI  **Substance use disorder**  **Anemia**  Digestive disease  Major mental health disorder  Gestational Age  **Length of stay**  Antepartum admission or ED visit  **Tobacco** **use**  SMM  Insurance  Delivery mode  **Nativity (foreign- vs. US-born)**  Late prenatal care intiation  ICE  **Distance to nearest delivery hospital (continuous, minutes)^a^** | 0.70 |

**Bold text** indicates candidate variables that were **not selected** in the final NYC prediction model.

^a^Not a candidate variable available for selection in NYC model.
